# Induction and assessment of persistent radioresistance in murine leukocytes *in vivo*

**DOI:** 10.1016/j.bbrep.2022.101296

**Published:** 2022-06-07

**Authors:** Pedro Morales-Ramírez, Virginia Cruz-Vallejo, Teresita Vallarino-Kelly, Regina Rodríguez-Reyes, Francisco González-Beltrán

**Affiliations:** Instituto Nacional de Investigaciones Nucleares, Mexico

**Keywords:** Radioresistance, Gamma rays, Comet assay, Mutation selection, Stem cells

## Abstract

The aim of the present study was to investigate whether weekly exposure to gamma rays causes a persistent increase in the number of radioresistant leukocytes in mice *in vivo*. Using the comet assay, 1 Gy radiation exposure decreased the percentage of leukocytes with less than 5% DNA in the tail (<5% DNAT), and we propose that radioresistance induction might increase the number of cells with <5% DNAT after radiation exposure. We exposed mice to 1 Gy gamma rays weekly for four weeks or 2 Gy per week for nine weeks. We observed a significant increase in cells with <5% DNAT after the third week and up to nine weeks of exposure. We exposed animals to gradually increasing radiation doses and finally challenged the lymphocytes with 1 Gy radiation both *in vivo* and *in vitro*. We observed increased radioresistance *in vitro*, providing evidence that a cellular process is involved. However, more radioresistance was observed *in vivo* than *in vitro*, suggesting a physiological effect. Cells challenged *in vitro* were maintained on ice during and after exposure, which likely caused a reduction in DNA repair. Radioresistance induction likely arose from mutation selection in stem cells because leukocytes are unable to proliferate in peripheral blood.

## Highlights

• First evidence of cell radioresistance induced *in vivo* in mice.
• Leukocyte precursor cells *in vivo* a model for study radioresistance induction.
• Irradiation-division cycles *in vivo* cause long-lasting cellular radioresistance.
• Increase of <5% DNA at tail after irradiation an index of cell radioresistance.
• Course of radioresistance caused by mutation-selection differ from adaptive response.

## 1 Introduction

Radioresistance is an intriguing phenomenon due to multiple varied factors that affect response thresholds of cells when receiving radiation. Living organisms are naturally exposed to very low doses of ionizing radiation from the environment. Ionizing radiation exerts its action mainly through the ionization of water and the formation of free radicals and oxidative species [1]. Cells have developed protection mechanisms since they normally generate free radicals during metabolism [2], so they are capable of neutralizing to a certain extent the action of radicals generated by ionizing radiation, through mechanisms of antioxidant activity [3]. Besides cells respond to the oxidative damage generated in the DNA, through repair mechanisms [4].

Nowadays, the extensive practice of radiotherapy in oncology has generated great attention in cellular radioresistance. Studies have identified radioresistant tumors that, when presenting highly malignant phenotypes, produce a poor prognosis [5].

Temporal cell radioresistance is induced by low-dose radiation exposure, which results in an increase in resistance at higher doses. This phenomenon is called adaptive response, and evidence indicates that this response is caused by short-term upregulation of DNA repair and antioxidant activities [6] and even occurs in human cells [7]. These adaptive response mechanisms appear shortly after conditioning radiation exposure and persist for approximately 24 h [8,9].

However, persistent radioresistance is induced in cells *in vitro* by mutations that affect different genes involved in the oxidative stress response [10,11], DNA repair [12,13] or apoptosis [14]. In bacteria, cycles of exposure to doses of UV radiation [15] or ionizing radiation [16] and growth induce mutations that confer radioresistance. This phenomenon seems to occur by a process in which radiation introduces variability by generating mutations and acts as a selective agent for adapted cells.

Recently, it has been reported that as people age, a substantial proportion of circulating blood cells in the hematopoietic system are derived from a single mutated stem cell. This process of mutation selection is called “clonal hematopoiesis” [17]. A similar process could be the origin of radioresistant cancer stem cells [18].

The aims of the present study were to develop an *in vivo* mouse assay to determine the increase in the number of radioresistant leukocytes and to determine whether weekly cycles of irradiation of mice *in vivo* induce an increase in the number of radioresistant leukocytes derived from the dual action of radiation as a mutagenic and selective agent on leukopoietic cells.

## 2 Materials and methods

### 2.1 Animals

Two-to three-month-old inbred albino male mice weighing approximately 30 g that descended from the BALB/c mouse strain were used in this study. The animals were maintained and bred in our laboratory under controlled environmental conditions with a temperature of 22 ± 3 °C, humidity of 60 ± 10% and dark-light periods of 12 h. The animals were fed Rodent Laboratory Chow 5001 for small rodents (PMI Nutrition International, Brentwood, MO, USA) and water *ad libitum*. Animals were treated and housed in accordance with the Committee for the Update of the Guide for the Care and Use of Laboratory Animals [19]. The study procedures were reviewed and approved by the Internal Committee of Care and Use of Laboratory Animals (CICUAL), which oversees the ethics of research involving laboratory animal use and welfare.

### 2.2 Reagents

Ethidium bromide, NaCl, EDTA, Trizma base, NaOH, N-lauryl-sarcosine, and Triton X100 were purchased from Sigma–Aldrich (Química, S de R.L. de C.V. Toluca, México). Agarose LMP and agarose were purchased from Gibco BRL, Life Technologies, Inc. (Gaithersburg, MD, USA).

### 2.3 Protocols

#### 2.3.1 Protocol I radioresistance induction with 1 Gy

For a group of 10 mice, whole-body exposure to 1.0 Gy ^60^Co gamma radiation was performed at the beginning of the experiment and once weekly for three weeks. Blood samples (4 μl) were obtained from the tail within 5 min after radiation exposure and placed on ice. Using a comet assay, the percentage of leukocytes with <5% DNAT was determined in one hundred cells before the first exposure (control) and after each radiation exposure. The data collected after the first radiation exposure represent the basal radioresistance.

**Table:** 

Protocol I		
Week	Radiation	Sample
0	R_0_ 0.0 Gy	S0
0	R_1_ 1.0 Gy	S1
1	R_2_ 1.0 Gy	S2
2	R_3_ 1.0 Gy	S3
3	R_4_ 1.0 Gy	S4

#### 2.3.2 Protocol II radioresistance induction with 2 Gy

For a group of 10 mice, whole-body exposure to 2.0 Gy ^60^Co gamma radiation was performed at the beginning of the experiment and once weekly for eight weeks. Blood samples (4 μl) were obtained from the tail within 5 min after radiation exposure and placed on ice. The percentage of leukocytes with <5% DNAT was determined in one hundred cells before the first irradiation dose (nonirradiated control) and after each subsequent radiation exposure. The data obtained after the first radiation exposure represent the basal radioresistance.

**Table:** 

Protocol II		
Week	Radiation	Sample
0	R_0_ 0.0 Gy	S0
0	R_1_ 2.0Gy	S1
1	R_2_ 2.0 Gy	S2
2	R_3_ 2.0 Gy	S3
3	R_4_ 2.0 Gy	S4
4	R_5_ 2.0 Gy	S5
5	R_6_ 2.0 Gy	S6
6	R_7_ 2.0 Gy	S7
7	R_8_ 2.0 Gy	S8
8	R_9_ 2.0 Gy	S9

#### 2.3.3 Protocol III confirmation of cellular radioresistance

A group of 10 animals was treated individually and subsequently subjected to acute exposure to doses of 1.0, 1.5 and 2.0 Gy of ^60^Co gamma radiation once per week for two weeks at each dosage.

**Table:** 

Protocol III			
Week	Radiation	Sample Irradiation *in vivo*	Sample Irradiation *in vitro*
0	R_0_ 0.0 Gy	S0_0_	
0	R_1_ 1.0Gy	S1	S1
1	R_2_ 1.0 Gy		
2	R_3_ 1.5 Gy		
3	R_4_ 1.5 Gy		
4	R_5_ 2.0 Gy		
5	R_6_ 2.0 Gy		
6	R_0_ 0.0 Gy	S0_6_	
6	R_7_ 1.0 Gy	S2	S2

Before the first 1.0 Gy exposure, 4 μl of blood were obtained from the tail and irradiated with 1.0 Gy while on ice. The other sample was used as a nonirradiated control. The mice then received the first dose of 1.0 Gy, and 4 μl of blood were obtained from the tail within 5 min after irradiation and maintained on ice. The data obtained after the first radiation exposure represent the basal radioresistance.

One week after the last 2.0 Gy conditioning exposure, two samples of 4 μl of blood were obtained from the tail. One sample was used as a nonirradiated control after conditioning, and the other was irradiated *in vitro* with a 1.0 Gy challenge dose while on ice. The mice then received a challenge dose of 1.0 Gy *in vivo*, and 4 μl of blood were obtained from the tail within 5 min after irradiation and maintained on ice.

**Fig. 1:**
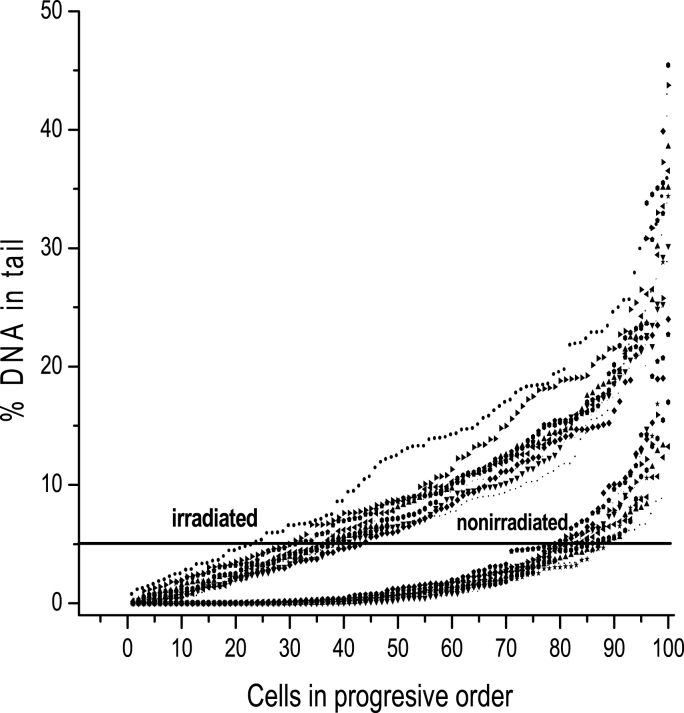
DNA in the tail from irradiated and nonirradiated leukocytes. Curves of DNA in the tails of leukocytes from nonirradiated mice and mice irradiated with 1.0 Gy of gamma rays. Each point represents a cell, and each curve represents 100 cells from each of ten mice. The cells are plotted in order of increasing tail DNA content. The line extrapolated from 5% DNA in the tail indicates that approximately 85% of the cells from the nonirradiated mice have less than 5% DNA in the tail (<5% DNAT) and that only approximately 30% of the cells from the irradiated mice have <5% DNAT.

Additionally, control blood samples were obtained before and after the first selective radiation exposure. The level of DNA damage and the percentage of leukocytes with <5% DNAT were determined in one hundred cells from all samples using a comet assay (Fig. 1).

#### 2.3.4 Samples

Blood samples were obtained from the tail by rapidly cutting the tip of the tail with small scissors, and then a 4-μl blood sample was obtained using an Eppendorf pipet. The first sample was considered the control sample for each mouse. The subsequent samples were collected immediately after irradiation.

#### 2.3.5 Irradiation

The mice were individually exposed to 1.0 or 2.0 Gy using a ^60^Co gamma ray source (Gammacell) at a dose rate of 0.9 Gy/min. The range of radiation doses used in this study has been shown to cause mutations [20] and a sufficient amount of DNA damage to be detectable by single-cell gel electrophoresis [8]. Radiation doses were confirmed by thermoluminescent dosimetry. During *in vitro* irradiation, blood samples were maintained in plastic tubes on ice.

#### 2.3.6 Alkaline single-cell gel electrophoresis assay

For single-cell gel assays, a previously described basic alkaline technique was used [21] with some modifications [22]. Briefly, 4-μl blood samples obtained from the tails were mixed with 100 μl of low-melting-point agarose (0.5%) and added to a slide with a dry layer of agarose. Then, the slides were exposed to an alkaline buffer (10 N NaOH, 1 mM EDTA) for 40 min. Next, an electric current of 25 V and 300 mA was applied for 40 min using a power supply (PS250-1, Techware, Sigma Chemical, St. Louis, MO, USA). This process was conducted under low light conditions to prevent additional DNA damage. The slides were removed, and Tris buffer (0.4 M Tris, pH 7.5) was added dropwise to neutralize the excess alkali solution. Then, the samples were rinsed thrice for 5 min each. The slides were then dehydrated in pure cold methanol and maintained in a closed box at room temperature. Prior to staining, the slides were rehydrated with Tris buffer. Each slide was stained with 50 μl of ethidium bromide (2.0 μg/ml) and covered with a clean coverslip. The slides were stored in a humidifier and evaluated less than 24 h after staining.

#### 2.3.7 Radioresistance index

In the present study, we scored the percentage of cells with <5% DNAT from 100 cells per mouse using the image analysis program Comet Assay IV (Perceptive Instruments, Inc., U.K.) and a fluorescence microscope equipped with an excitation filter of 515–560 nm, a bar filter of 590 nm and a 25X objective.

The percentage of radioresistant cells was determined by detecting an increase in cells with <5% DNAT, as scored by alkaline electrophoresis in whole blood after exposure to 1.0 Gy of gamma rays *in vivo*, where each radiation exposure permits the determination of the percentage of radioresistant cells at the time of exposure and could induce mutations and variability for the next week.

#### 2.3.8 Statistics

Because samples were collected after the first exposure to radiation in each mouse, these samples were used as controls of basal radioresistance for each mouse. This design permits statistical comparisons with the control samples using both paired and unpaired t tests (significance defined by p < 0.05). The paired *t*-test increases the statistical power by accounting for random variation occurring between animals. Statistical analyses were performed with Microsoft Excel *(Office)*.

## 3 Results

An experiment was completed to establish an index of radioresistance. Fig. 1 compares the curves representing the percent of DNA in the tail per cell from 100 cells collected from each of 10 mice before and after treatment with 1.0 Gy of gamma rays (^60^Co). The data per cell are shown in increasing order of DNA in the tail. A total of 85% of cells from the control mice had less than 5% DNA in the tail (<5% DNAT), whereas only 30% of cells from 1.0 Gy-irradiated mice had <5% DNAT. Therefore, a reasonable hypothesis is that if cells acquire radioresistance, an increase in the number of cells with <5% DNAT would be observed after irradiation.

**Fig. 2:**
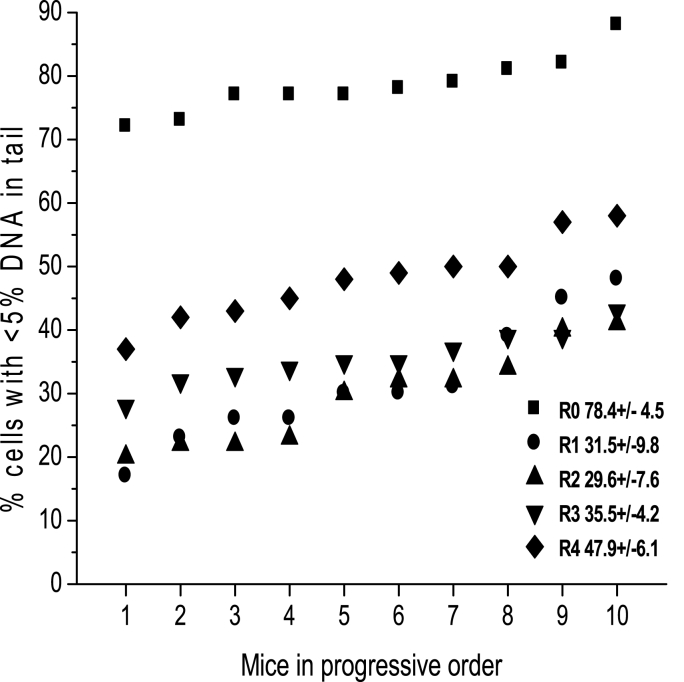
Percentage of leukocytes with <5% DNAT after weakly 1.0 Gy exposures. The percentage of murine leukocytes with <5% DNAT sampled before irradiation and immediately after the first and each of three subsequent weekly exposures to 1.0 Gy of gamma rays. The mice were ordered according to the frequency of cells with <5% DNA in the tail. After four exposures, the response was significant (p < 0.05, Student's *t*-test) compared to the first radiation exposure. Each exposure served as a challenge dose immediately after irradiation and as a selective dose for subsequent exposures.

The same animals were sampled before treatment and after the first and subsequent weekly irradiation regimens described in Protocol I to establish whether mutagenic-selective radiation doses of 1.0 Gy increase radioresistance and whether this resistance persists. Blood samples were acquired 5 min after irradiation, and the frequency of cells with <5% DNAT was determined. Fig. 2 shows the frequency of cells with <5% DNAT per mouse in sequential order based on samples that were obtained after each exposure. The results indicate an increase in the number of cells with <5% DNAT in the animals exposed once per week to 1.0 Gy, and this increase was statistically significant after four exposures with respect to the first exposure.

**Fig. 3:**
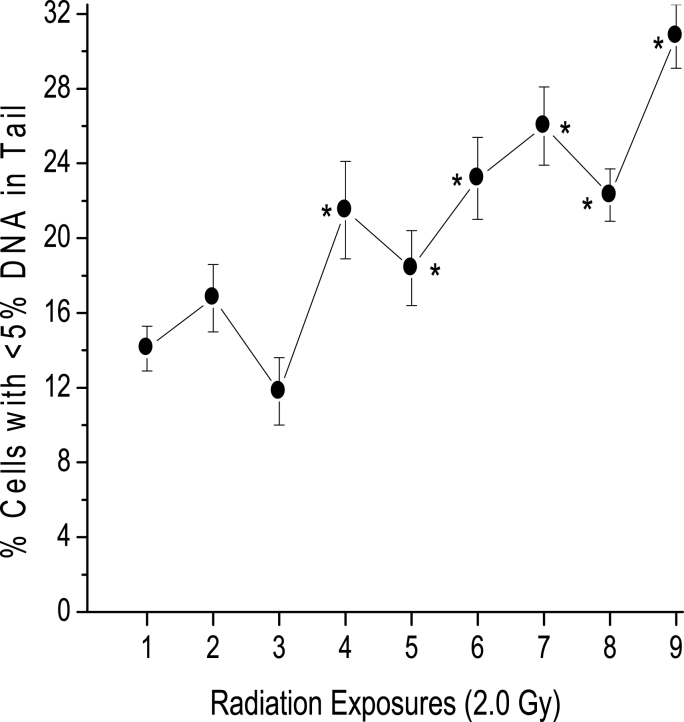
Percentage of leukocytes with <5% DNAT vs. number of exposures to 2.0 Gy. The mean percentage and SE of murine leukocytes with <5% DNAT sampled in ten mice before irradiation and immediately after the first and each of the weekly acute exposures to 2.0 Gy of gamma rays. From the fourth exposure to the last, the responses were statistically significant (p < 0.05, Student's *t*-test) compared with the first exposure frequency, and significant points are indicated using an asterisk.

In a second experiment, mice were treated with 2.0 Gy weekly for 9 weeks to explore whether the frequency of cellular radioresistance increases with a higher dose and number of radiation treatments (Protocol II). The results are shown in Fig. 3. The data indicate that the frequency of cells with <5% DNAT increased significantly with respect to the first irradiation after the fourth exposure and further increased after nine exposures. Although irradiation with 2.0 Gy caused a greater initial reduction in the population of cells with <5% DNAT, the overall increase in the percentage of cells with <5% DNAT for 9 weeks (17%) was similar to that obtained with 1.0 Gy for 4 weeks (18%). The curve indicates a tendency for the number of radioresistant cells to increase after subsequent radiation exposure. Fewer cells were obtained for analysis after seven exposures, indicating that radiation exerted a detrimental effect on the animals.

Protocol III enabled us to determine whether the cells challenged *in vivo* were as radioresistant as the cells challenged *in vitro* to document the effect at the cellular level. The irradiation protocol was modified to reduce the deleterious effects of radiation by exposing the cells to progressive radiation doses and to eliminate a possible effect of continuous sampling by sampling only at the beginning and at the end of the experiment. The last radiation challenge dose was administered one week after the six mutation-selective radiation exposures.

**Fig. 4:**
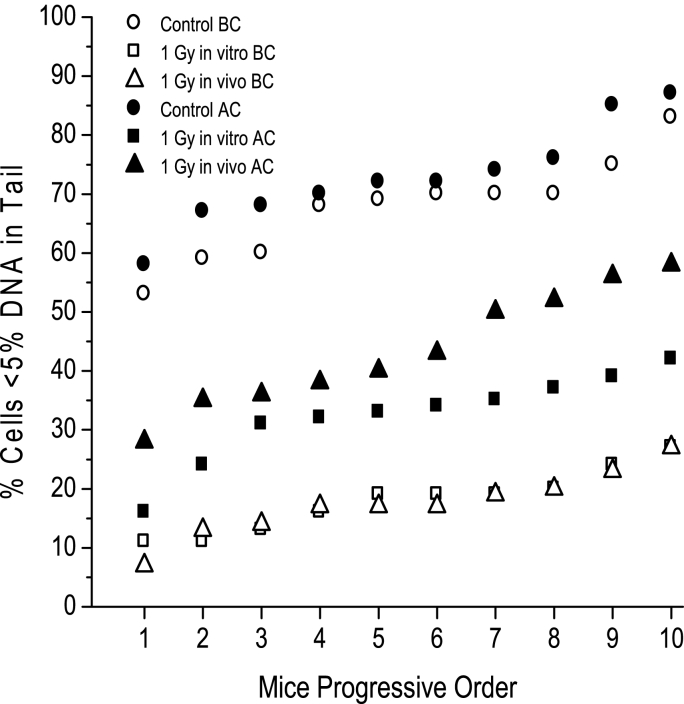
Percentage of leukocytes with <5% DNAT *in vivo* and *in vitro* after six conditioning radiation exposures. Percentage of murine leukocytes with <5% DNAT exposed to 1.0 Gy before (BC) and after (AC) the conditioning protocol of weekly exposure to 1.0, 1.5 and 2.0 Gy for two weeks each (Fig. 1). The percentage of leukocytes with <5% DNAT was determined in peripheral blood leukocytes irradiated either *in vivo* or *in vitro* with 1.0 Gy one week after exposure to the selective dose. No difference in the unirradiated controls was observed before and after the conditioning exposures, and no difference was observed after 1.0 Gy exposure *in vivo* or *in vitro* before the mutation selection protocol. Selection treatment significantly increased the percentage of leukocytes with <5% DNAT both *in vivo* and *in vitro* compared with samples from nonconditioned mice (p < 0.05, Student's *t-test*).

Approximately the same percentage of cells with <5% DNAT was observed in nonirradiated control cells before and after the selection protocol (Fig. 4). The cells exposed to 1.0 Gy radiation *in vivo* and *in vitro* before the mutation selection protocol showed the same degree of reduction in the population of cells with <5% DNAT. After irradiation, a significant increase in the percentage of cells with <5% DNAT was observed in the groups challenged with 1.0 Gy *in vivo* and *in vitro*. A significantly higher percentage of cells challenged *in vivo* after the irradiation protocol had <5% DNAT than the cells challenged *in vitro* (Table 1). The overall increase in the percentage of cells with <5% DNAT with this protocol *in vivo* was 26%.

**Table 1:** Percent of radioresistent leukocytes after 1.0 Gy challenge IN VIVO and in vitro, before (BC) and after (AC) conditioning treatment.

	% Cells <5%_DNA_T x ± SD			
	Control	1 Gy *in vivo*	1 Gy *in vitro*	Mice
Before Conditioning (BC)	67.7 ± 8.5	17.4 ± 5.5^a^	17.9 ± 5.3^a^	10
After Conditioning (AC)	72.9 ± 8.5	43.6 ± 9.9^a,b,c^	31.9 ± 7.0^a,b,c^	10

a Significant *vs* control*.*

b Significant AC *vs* BC.

c Significant AC *in vivo vs* AC *in vitro*, with both paired ***t***-test and Students ***t***-test, p < 0.05.

## 4 Discussion

Several parameters have been used in previous studies to measure radioresistance, but the most common include increased viability [23] or an increased capacity for DNA damage repair [24] and a direct decrease in apoptotic response [25]. These parameters are not easily assayed *in vivo* immediately after radiation exposure at the cellular level. The percentage of DNA in the tail is particularly useful because it allows us to measure DNA damage in each cell. This technique allows us to establish a limit for considering damaged and undamaged cells by comparing the curves of damaged cells from irradiated and nonirradiated mice. The frequency of cells with <5% DNA T was a good index because the percentage of cells with <5% DNAT was approximately 85% in untreated mice and was reduced to 30% after exposure to 1.0 Gy of radiation. Under these circumstances, the increase in the number of cells with <5% DNAT after radiation exposure resulted in a clear index of radioresistance.

We proposed that a procedure including subsequent irradiation and division periods would cause cell death, which stimulates cell division in leukocyte precursors or stem cells in the bone marrow and might also induce a mutation-selection process that increases the number of radioresistant cells. Recently, mutation selection was shown to normally occur in human hematopoietic cells. This process represents the clonal hematopoiesis phenomenon observed in elderly human populations caused by a mutation selection process [17]. According to data previously published, the radiation dose used in our protocol is able to increase the mutation rate [20] and acts as a selective agent by killing more sensitive cells [26]. This result is similar to the radioresistant *Escherichia coli* obtained after experimental evolution with 100 cycles of mutation-selection with ionizing radiation [16].

Neutrophils and lymphocytes are the most prevalent white blood cells in peripheral blood, and published evidence has indicated that human peripheral blood cells exhibit different radiosensitivities. Monocytes and granulocytes are more radioresistant than lymphocytes [27]. In our experiments, the possibility of selection without mutation is very likely to occur, but the increase in the percentage of resistant cells would reach a maximum and would fluctuate because 14 cell generations are produced between each period of irradiation, assuming 12 h as the average generation time [28]. Fig. 3 shows that the resistance increases slightly in the 2 nd week but decreases in the third week. This behavior would probably be repeated in the absence of mutation; however, there is an increasing trend with respect to baseline from the 4th week in animals treated with 1.0 and 2.0 Gy, which suggests the incorporation of radioresistant lineages.

Neutrophils and lymphocytes continuously turn over in peripheral blood [29,30], and *in vivo* experiments in mice have shown that an acute dose of irradiation results in cell death, which promotes a proliferative homeostatic process in bone marrow to recover cell numbers [31]. The hematopoiesis in adult mice occurs mainly in the bone marrow [32], which implies that the mutation-selection process might occur in precursor cells or stem cells in the bone marrow [33]. Thus, our results indicate that the induction of persistent radioresistance in leukocytes was probably due to the selection or mutation selection of precursor or stem cells, which generates radioresistant cell lineages [34]. Eukaryotic cells have developed strategies to ameliorate genetic damage caused by free radicals, which are useful for the damage induced by ionizing radiation. These strategies imply an increase in the efficiencies of DNA repair [34] and activities that reduce oxidative stress [35].

Bone marrow stem cells are heterogeneous in terms of radioresistance, suggesting the possibility that our protocols of radiation exposure select the radioresistant fraction of existing stem cells [36]. Thus, the possible involvement of stem cells in emergent radioresistant cells must be considered. For example, cancer stem cells proliferate and subsequently produce the majority of differentiated cancer cells [37]. This phenomenon has been observed in leukemia [38] and other types of cancer [39]. Many studies of radioresistance at the cellular level have been performed in cancer cells because tumors containing cancer stem cells are highly malignant and are associated with a poor response to conventional radiotherapy and chemotherapy [40].

The measurement of the response each week during weekly selective irradiation with 1.0 or 2.0 Gy revealed weekly persistent radioresistance that was induced from the fourth week onward and increased at least up to nine weeks of exposure. This persistent radioresistance was induced by a mechanism that differed from those responsible for the adaptive response given that stimulation of the adaptive response *in vivo* was observed in leukocytes 60 min after exposure to doses as low as 0.01 Gy and persisted for only approximately 24 h [8,9].

Because the experiments presented here were conducted *in vivo*, the observed resistance was potentially due to extracellular radioprotection, i.e., an increase in selenium proteins [41] or glutathione [42] in the blood, which may protect cells and the organism from free radicals. This possibility was examined by challenging leukocytes isolated *in vitro* from *in vivo*-treated mice with the selection protocol. The isolated cells were radioresistant, suggesting that radioresistance was a cellular phenomenon. However, because the resistance of cells challenged *in vivo* was greater than that of cells challenged *in vitro*, a physiological phenomenon potentially occurred.

The present study provided the first evidence of cell radioresistance induced *in vivo* and established the basis of a model to study stem cell renewal and differentiation. The experimental model presented here might facilitate the exploration of several aspects of radioresistance. Leukocyte precursor cells appear to be a convenient biological model for the study of radioresistance because they are continuously dividing, and descendant leukocytes are easy to obtain via the mouse tail, allowing the radioresistance of individual cells in peripheral blood to be monitored without requiring significantly invasive procedures.

The analysis of the experimental results allowed us to generate the following conclusions:

i) The present study provided evidence that successive periods of exposure to irradiation and cell division *in vivo* induce long-lasting cellular radioresistance.
ii) The percentage of cells with <5% DNAT, as estimated by single-cell gel electrophoresis, may represent an appropriate index of cell resistance after radiation exposure.
iii) Successive radiation exposure in mice causes peripheral leukocytes to gradually become radioresistant *in vivo.*
iv) The mechanism of radioresistance induced in the present study differs from the adaptive response observed *in vivo* given that its stimulation required higher doses, appeared after the fourth week of radiation exposure and persisted for at least one week. The adaptive response requires low doses, appears almost immediately and persists for only 24 h.
v) This radioresistance induction likely involves precursor or stem cells because leukocytes are unable to proliferate in peripheral blood.
vi) The experimental model developed in the present study will enable the exploration of the mechanisms by which long-lasting radioresistance is induced in murine leukocyte populations and in individual cells *in vivo.*

## Statement of authors’ contributions

All authors have made substantial contributions to the conception and design of the study, acquisition of data, analysis and interpretation of data, drafting the article and revising it critically for important intellectual content, and approval of the version submitted.

## Funding source

This work was supported by a project CB-211 of the Instituto Nacional de Investigaciones Nucleares (ININ). This research did not receive any specific grant from funding agencies in the public, commercial, or not-for-profit sectors.

## Declaration of competing interest

Pedro Morales-Ramírez, Virginia Cruz-Vallejo, Teresita Vallarino-Kelly, Regina Rodríguez-Reyes, Francisco González-Beltrán confirm that they have any conflicts of interest to declare.

## Data Availability

Data will be made available on request.
